# The People and the Code

**Published:** 1884-05-20

**Authors:** 


### THE PEOPLE AND THE CODE.

The object, we suppose, of the suggestion of Dr. Flint, that the American Medical Association adopt a resolution explaining more particularly the reasons for not consulting with irregular practitioners, is to enlighten the people on the subject. The common people do not understand, and seem to have no patience with what they regard as mere punctiliousness, as between physicians in the matter of refusing consultations with members of different schools of medicine. This is largely due to the republican ideas which have ever been the boast of our people, and which they instintcively apply to every calling and to every department of life.

There are at this time petitions before Congress from the States of Kentucky, South Carolina, Connecticut and New Jersey, protesting against the discriminations which are made in the Army, Navy and Civil Service appointments against certain schools of medicine.

